# Draft Genome Sequences of Eight Pseudomonas aeruginosa Corneal Infection Isolates

**DOI:** 10.1128/MRA.01253-19

**Published:** 2020-01-02

**Authors:** Vishnu Raghuram, Joanna B. Goldberg

**Affiliations:** a Microbiology and Molecular Genetics Program, Graduate Division of Biological and Biomedical Sciences, Laney Graduate School, Emory University, Atlanta, Georgia, USA; b Department of Pediatrics, Division of Pulmonary, Allergy and Immunology, Cystic Fibrosis, and Sleep, Emory University School of Medicine, Atlanta, Georgia, USA; c Emory Children’s Center for Cystic Fibrosis Research, Emory University and Children’s Healthcare of Atlanta, Atlanta, Georgia, USA; Loyola University Chicago

## Abstract

Pseudomonas aeruginosa is the major cause of bacterial keratitis, a sight-threatening ocular infection that can occur in contact lens wearers, as well as in others. Here, we report the draft genomes of 8 different P. aeruginosa corneal isolates, adding to the list of publicly available corneal infection-associated P. aeruginosa genomes.

## ANNOUNCEMENT

Pseudomonas aeruginosa is a Gram-negative opportunistic pathogen that causes infections in immunocompromised people. While well known for its ability to cause infections of the respiratory tract, P. aeruginosa is relatively understudied in the context of bacterial keratitis (infection of the cornea). P. aeruginosa is the major cause of bacterial keratitis, occurring in 71% of all infection-positive contact lens wearers (1, 2). Electron microscopic studies have shown that P. aeruginosa adheres to and penetrates corneal epithelial cells (3). A number of exo-enzymes secreted by P. aeruginosa, such as PrpL, PASP, ExoS, ExoT, and ExoU, are associated with increased virulence and can cause corneal damage, potentially leading to permanent vision loss (4–7). However, the exact mechanisms responsible for P. aeruginosa-associated eye infections are not fully understood.

There is a need for more corneal isolates in the pool of sequenced P. aeruginosa strains. The Pseudomonas Genome Database (8) has over 4,800 genomes, out of which only 2 are reported to be corneal isolates. Here, we report the genome sequence of 8 additional P. aeruginosa isolates collected from human eye infections by the Bascom Palmer Eye Institute (Miami, FL) (9, 10), contributing to the diversity of sequenced P. aeruginosa clinical isolates. Our goal is to identify genetic features common to corneal isolates and in the future understand the roles that particular factors play in corneal virulence.

All isolates were incubated at 37°C overnight in 3 ml lysogeny broth (LB) with shaking. Genomic DNA was isolated using a Qiagen DNeasy blood and tissue kit. Samples were then sequenced using an Illumina NextSeq 500 instrument (Microbial Genome Sequencing Center, University of Pittsburgh), producing 151-bp paired-end reads. Sequencing libraries were prepared by the sequencing center as described by Baym et al. (11). Quality trimming was performed using Trim Galore! v0.6.2, and reads with a quality score of >27 were selected for assembly using SKESA v2.3.0 (12–14). These draft assemblies were then fed into SPAdes v3.13.1 with the “–trusted-contigs” parameter, along with reads having a quality score of >20 as the input (15). The “–cov-cutoff” parameter in SPAdes was set to “auto.” Quality assessment for the resulting assemblies was performed using QUAST v5.0.2 (16). The assemblies were then annotated using NCBI Prokaryotic Genome Annotation Pipeline v4.9 (17). Default parameters were used for all software unless otherwise noted.

The final assemblies have an average coverage of 80×, with 110 contigs and 66.2% GC content. All strains were positive for the exo-enzymes PrpL, PASP, and ExoT. All strains except 6487 were positive for ExoU, and only strain 6487 was positive for ExoS.

### Data availability.

The accession numbers and assembly statistics of these isolates are listed in Table 1. The BioProject accession number is PRJNA558357.

**TABLE 1:** Genome assembly statistics of 8 P. aeruginosa isolates recovered from human corneal infections

Strain name	GenBank accession no.	SRA accession no.	No. of raw reads	No. of contigs	Total length (bp)	GC (%)	*N*_50_ (bp)	*L*_50_	Assembly coverage (×)
6073	VOLA00000000	SRR9904145	2,110,378	154	6,920,359	66.1	97,264	18	75
6206	VOKZ00000000	SRR9904146	2,375,999	121	6,901,724	66.05	138,064	19	85
6354	VOJG00000000	SRR9904143	1,786,529	89	6,334,363	66.51	136,410	14	72
6382	VOJF00000000	SRR9904144	2,777,128	95	6,539,906	66.32	144,880	17	109
6389	VOKY00000000	SRR9904149	2,243,631	110	6,537,335	66.31	112,407	17	84
6436	VOJE00000000	SRR9904150	2,058,162	120	6,700,809	66.16	115,309	17	77
6452	VOJD00000000	SRR9904147	2,475,116	109	6,743,767	66.1	141,768	17	90
6487	VOJC00000000	SRR9904148	2,110,378	86	6,338,660	66.53	114,717	17	74
